# Multisystem Inflammatory Syndrome in Children, the Real Disease of COVID-19 in Pediatrics - A Multicenter Case Series From Al-Ahsa, Saudi Arabia

**DOI:** 10.7759/cureus.11064

**Published:** 2020-10-20

**Authors:** Zainab A Almoosa, Heba H Al Ameer, Sajjad M AlKadhem, Fadi Busaleh, Fatimah A AlMuhanna, Osama Kattih

**Affiliations:** 1 Pediatric Infectious Diseases, Almoosa Specialist Hospital, Hofuf, SAU; 2 Infectious Diseases, Maternity and Children Hospital, Al-Ahsa, SAU; 3 Pediatrics, Maternity and Children Hospital, Al-Ahsa, SAU; 4 Pediatric Intensive Care Unit, Almoosa Specialist Hospital, Al-Ahsa, SAU

**Keywords:** covid-19, msi-c, kawasaki, toxic shock syndrome

## Abstract

Fortunately, coronavirus disease 2019 (COVID-19) infection in pediatric populations exhibits a mild course of disease. However, a small number have recently been identified who develop a significant systemic inflammatory response, a new disease entity called multisystem inflammatory syndrome in children (MIS-C), especially after the peak of the wave in Al-Ahsa, Saudi Arabia, in early June to mid-July. In MIS-C children usually present a few days to a few weeks after recovery from COVID-19 with high grade fever, GI symptoms, Kawasaki-like picture or even toxic shock-like syndrome. Raising awareness about this disease entity is very fundamental to enable pediatricians and other health care providers to identify and manage these patients before it is too late. We describe 10 different cases of MIS-C with different risk factors and presentations.

## Introduction

As severe acute respiratory syndrome coronavirus 2 (SARS-CoV-2) was announced by the World Health Organization (WHO) to be a pandemic early in March 2020, the main concern was about the respiratory disease in adults, as affected pediatric cases were asymptomatic or had mild symptoms as initial infection [1]. Later, in May 2020, Riphagen et al. published a case series of eight children who presented with a Kawasaki shock-like picture, but it was linked to SARS-Cov-2 infection as a latent infection effect [2]. Such presentations were noticed later across the globe and raised the attention for the possibility of a latter effect of SARS-Cov-2. This Kawasaki shock-like presentation, which was concurrent with SARS-Cov-2, was named by the WHO as “multisystem inflammatory syndrome in children and adolescents temporally related to COVID-19” (MIS-C) [3-4]. MIS-C is characterized by multiorgan involvement, mainly gastrointestinal symptoms with cardiovascular collapse, with an increase in inflammatory markers and a history of recent infection or contact with COVID-19 cases [4]. There is no absolute definition for MIS-C, but as time progresses, our knowledge is evolving. This article describes a case series of MIS-C from two centers in Al-Ahsa, Saudi Arabia.

## Materials and methods

Hospitals in Al-Ahsa, Saudi Arabia reported cases of Kawasaki disease, toxic shock syndrome, and MIS-C in hospitalized patients younger than 14 years of age. We carried out descriptive analyses that summarized the clinical presentation, complications, and outcomes of patients who met the case definition for MIS-C between June 1 and July 30, 2020.

We collected 10 cases from two centers in Al-Ahsa, Saudi Arabia. The diagnosis was made based on the Centers for Disease Control and Prevention (CDC) case definition: 1. An individual aged <21 years presenting with fever, laboratory evidence of inflammation, and evidence of clinically severe illness requiring hospitalization, with multisystem (>2) organ involvement (cardiac, renal, respiratory, hematologic, gastrointestinal, dermatologic, or neurological), 2. No alternative plausible diagnoses, and 3. Positive for current or recent SARS-CoV-2 infection by reverse-transcriptase polymerase chain reaction (RT-PCR), serology, or antigen test; or exposure to a suspected or confirmed COVID-19 case within the four weeks prior to the onset of symptoms.

Evidence of inflammation means one or more of the following: an elevated C-reactive protein (CRP), erythrocyte sedimentation rate (ESR), fibrinogen, procalcitonin, d-dimer, ferritin, lactic acid dehydrogenase (LDH), or interleukin 6 (IL-6), elevated neutrophils, reduced lymphocytes, and low albumin.

## Results

Cases

First Case

A 13-year-old girl known to be glucose-6-phosphate dehydrogenase (G6PD) deficient was COVID-19 positive 20 days before the presentation. She presented to the emergency department (ED) with a high-grade fever for five days accompanied by lethargy, abdominal pain, diarrhea, maculopapular skin rash, bilateral non-suppurative conjunctivitis, and erythematous cracked lips. On the second day of hospitalization, she deteriorated with a shock-like picture: tachypnea, tachycardia, and hypotension, with delayed capillary refill time, and decreased level of consciousness. The patient was shifted to the Pediatric Critical Care Unit (PICU) with impression of MIS-C. She developed electrolyte disturbances (hyponatremia, hypokalemia), coagulopathy with high inflammatory markers, leukocytosis, and high cardiac enzymes. Echocardiography showed depressed left ventricular function, mitral valve regurgitation, and mild pericardial effusion. The patient was started on antibiotics and antiviral therapy: favipiravir, tocilizumab, low molecular weight heparin (LMWH), intravenous immunoglobulin (IVIG), and methylprednisolone. She was also kept on vasopressors, but she was deteriorating rapidly in the form of respiratory failure and persistent metabolic acidosis, acute respiratory distress syndrome (ARDS), and multi-organ failure. On the sixth day of admission to PICU she was declared dead after refractory hypotension secondary to myocardial dysfunction and cardiac arrest.

Second Case

A seven-year-old boy medically free presented to ER with five days history of fever, abdominal pain, and vomiting. He was also found to have bilateral non-purulent conjunctivitis, cracked lips, and cervical lymphadenopathy. COVID-19 PCR was positive with a history of exposure to COVID-19 patients two weeks prior to presentation. Laboratory investigations were suggestive of multi-organ failure with high inflammatory markers. He was admitted to PICU, where a chest X-ray showed ARDS picture and echocardiography showed pericardial effusion. The patient was started on IVIG, antibiotics, and antiviral therapy: favipiravir, LMWH, and dexamethasone. He received inotropic support and was connected to a mechanical ventilator. Unfortunately, the patient deteriorated rapidly with persistent hypotension, pulmonary hemorrhage, and coagulopathy, and was declared dead due to cardiac arrest.

Third Case

A seven-year-old girl known to be G6PD deficient presented to the ED with a three-day history of fever, irritability, lethargy, myalgia, maculopapular skin rash, and abdominal pain. She was admitted to PICU with impression of MIS-C. COVID-19 PCR was positive. She was started on IVIG and antibiotics as well as dexamethasone. Echocardiogram, ultrasonography, and X-ray were all within normal. She showed improvement after IVIG and discharged home after six days after admission in good condition.

Fourth Case

An 11-year-old boy, with known Diabetes Mellitus type 1, came with history of fever, vomiting, and abdominal pain for three days followed by eruption of generalized maculopapular skin rash with erythematous cracked lips and bilateral non-purulent conjunctivitis. Upon examination, he was febrile and tachycardiac but maintained blood pressure. There were palpable cervical and inguinal lymphadenopathy. He was admitted to the PICU as a case of atypical Kawasaki disease. The initial laboratory investigations showed anemia with high ESR and hyponatremia. Both chest X-ray and echocardiogram were normal. Later, the patient became hypotensive, normal saline bolus was given, and inotropic support started. He also developed leukocytosis and hypoalbuminemia. The constellation of signs and symptoms led to the diagnosis of MIS-C. The patient was started on IVIG, antibiotics, and steroids. He improved and was discharged home in good condition.

Fifth Case

A three-year-old girl, medically free, came with history of persistent fever for five days, associated with fatigue and diarrhea. She also developed generalized maculopapular skin rash and bilateral non-purulent conjunctivitis. She was COVID-19-positive three weeks prior to presentation. Upon examination, patient was lethargic, hypoactive, febrile, and hypotensive with generalized maculopapular rash. Patient was resuscitated with normal saline boluses and admitted to PICU with impression of MIS-C. Labs showed significant hypoalbuminemia and high LDH with hyponatremia. She was started on antibiotics and antiviral therapy (favipiravir). She was kept on inotropic support and started on pulse methylprednisolone, followed by IVIG. Her chest X-ray and echocardiography were normal. Patient showed a significant improvement after IVIG and was weaned off inotropes and fever subsided. She was discharged home in good condition on steroids tapering dose over four weeks.

Sixth Case

A one-year-old boy, medically free, presented with a one-week history of high-grade fever, associated with vomiting, diarrhea, and decreased oral intake with significant hypo-activity. Later, he developed a generalized skin rash and bilateral non-purulent conjunctivitis. There was a history of exposure to a positive case of COVID-19 about one month back. Upon examination, the patient was lethargic, febrile, with generalized maculopapular rash, bilateral non-purulent conjunctivitis, and cracked erythematous lips. There was a palpable cervical lymph node and limb edema. Laboratory investigations showed elevated inflammatory markers, anemia, and thrombocytopenia. Patient was admitted to the PICU as a case of Kawasaki disease vs MIS-C. He was started on IVIG followed by pulse methylprednisolone and aspirin. He also received favipiravir and LMWH. Patient improved significantly and discharged home in good condition. Echocardiography was normal.

Seventh Case

A 12-year-old girl had just recovered from a mild COVID-19 infection three weeks ago. All other family members were infected also. Her symptoms at that time were mild headache, low-grade fever, and muscle pain. She presented this time with a five-day history of high-grade fever, abdominal pain, and diarrhea. She was admitted initially as a case of suspected appendicitis. Subsequently, she developed generalized skin rash and non-purulent conjunctivitis. She denied any history of cracked lips or skin peeling.

Upon reviewing her history, she developed Kawasaki disease at age of four months which was complicated by coronary aneurysm.

On examination she was febrile (40.1°C) and tachycardic. Respiration and blood pressure were within normal limits. Non-purulent conjunctivitis and a diffuse reticular, papular rash were noticed. No cracked lips or strawberry tongue, hand edema, or skin peeling were noticed.

Laboratory tests showed leukopenia with left shift and lymphopenia. Platelets were normal. Inflammatory markers were high. D-dimer was extremely elevated. Echocardiography was normal.

She met the case definition of MIS-C both clinically and laboratory. IVIG was initiated, followed by methylprednisolone. She was also kept on low-dose aspirin and antibiotics. Fortunately, she showed significant improvement with complete clinical resolution within a few days.

Eighth Case

A six-year-old girl presented who was previously healthy. All family members including her were COVID-19-positive and recovered two weeks prior to presentation. The child presented with high-grade fever and diarrhea for five days. She visited ED two times where COVID-19 PCR was done and it was positive, and she was discharged home as a case of simple COVID-19 infection. Because of the persistence of symptoms, she was finally admitted with impression of MIS-C.

Upon examination she was ill, toxic looking, confused, febrile (40°C), and tachycardic. Respiration and blood pressure were normal. Cracked lips, non-purulent conjunctivitis, erythematous skin rash mainly over medial proximal lower limbs, and hand edema were noticed.

Laboratory tests showed mild thrombocytopenia, electrolyte imbalance, hyponatremia and hypokalemia, and metabolic acidosis. Ferritin was high in addition to hypophosphatemia, hypocalcemia in addition to hypoalbuminemia. Troponin and B-type natriuretic peptide (BNP) were elevated.

She deteriorated and developed signs of shock with hypotension despite giving boluses of normal saline and vasopressors. She was transferred to the PICU where she received IVIG, aspirin, antibiotics, pulse methylprednisolone, and tocilizumab. She showed significant improvement.

She stayed in the PICU for seven days before she was discharged home in good condition. Echocardiogram was normal.

Ninth Case

A five-year-old boy, a known case of sickle cell trait, presented with a five-day history of high-grade fever which was associated with abdominal pain. He also developed eye redness and skin rash. Three weeks before, he had been exposed to his grandmother who was COVID-19 positive. He was not tested for COVID-19 and he did not develop any symptoms at that time.

On examination: he was well-looking, febrile (39.5°C), and blood pressure was low (79/46). Non-purulent conjunctivitis, unilateral cervical lymphadenopathy, and a generalized maculopapular rash were noticed. He had cracked lips with strawberry tongue, hand, and feet edema as well as skin peeling. 

Laboratory investigations showed normal white blood cells (WBCs) and platelets of 230 which later increased up to 560 and high inflammatory markers. Hypoalbuminemia and electrolytes imbalance mainly hypernatremia, ferritin was elevated, and D-dimer was high. COVID-19 PCR was negative but COVID serology (IgG) was positive. The viral respiratory panel came back positive for influenza B.

He was admitted to PICU with an impression of MIS-C and given IVIG and methylprednisolone. Echocardiography showed left ventricular dysfunction. He was started on aspirin and he received oseltamivir for influenza. He improved significantly and was discharged home in good condition on aspirin. After two weeks from discharge, echo was repeated and it showed persistent left ventricular dysfunction, he was started on captopril and Lasix.

Tenth Case

An 11-year-old boy, a known case of G6PD deficiency, presented to ED with history of high-grade fever, vomiting, and diarrhea for three days duration. Both parents were COVID-19 positive a month ago. He was admitted initially to the regular ward, where he was found to be continuously febrile with abdominal pain. He also developed generalized skin rash as well as eye redness.

Upon examination, he was lethargic, pale, febrile (39°C), and hypotensive; blood pressure was 86/42 and he was tachypneic. He had non-purulent conjunctivitis, generalized skin rash, cracked lips, and strawberry tongue. Abdominal examination showed generalized tenderness.

Laboratory investigations showed anemia, thrombocytopenia, hyponatremia, hypophosphatemia, hypocalcemia in addition to hypoalbuminemia. Troponin and BNP were elevated. D-Dimer was elevated. He had very high ferritin. Inflammatory markers were elevated. COVID-19 PCR was negative but with positive COVID-19 serology IgG.

He was admitted to PICU with an impression of MIS-C where he was given boluses of IV fluids, IVIG, and methylprednisolone. Echocardiography was normal. He improved after a few days and was discharged in good condition.

We have reported on 10 patients with MIS-C in two hospitals. Nine (90%) were positive for COVID-19 by RT-PCR or antibody testing. Gastrointestinal system involvement was found in all 10 patients (100%) while 30% of the cases had cardiovascular involvement with almost half receiving vasopressor or vasoactive support. Patients presented with Kawasaki-like features as well as a shock-like picture. One of the patients was known to have Diabetes Mellites type 1 and three of them had G6PD deficiency (Table 1). Regarding laboratory investigations: four (40%) patients had leukocytosis and five (50%) showed lymphopenia. All 10 patients (100%) developed acute anemia secondary to MIS-C, while four patients (40%) developed thrombocytopenia. Two patients (20%) developed abnormal renal function test while seven patients (70%) had high liver enzymes, either alanine aminotransferase (ALT) or aspartate aminotransferase (AST). Most patients developed electrolytes imbalance: nine (90%) hyponatremia, eight (80%) hypocalcemia, and four (40%) hypokalemia. All patients (100%) developed significant hypoalbuminemia. The majority of patients (90%) had elevations in inflammatory markers, either ESR or CRP. Nine patients (90%) had high LDH and eight (80%) had high ferritin. Seven (70%) of the patient developed an abnormal coagulation profile (Table 2). Three patients (30%) had left ventricular dysfunction and pericardial effusion but coronary-artery aneurysms were not documented in any of the patients. Seven patients (70%) had an abnormal abdominal ultrasound (US) with either ascites or mesenteric adenitis (Table 3). Ten patients (100%) received intensive care, two (20%) received mechanical ventilation, five (50%) received vasoactive support. Specific antiviral treatment (favipiravir) was given to seven patients (70%). All patients received heparin, antibiotics, intravenous immune globulin, and steroids. Most of the patients (80%) survived and 20% died (Table 4). The two mortalities had no previous known risk factors except one of them was G6PD deficient.

**Table 1:** Clinical Data COVID-19: coronavirus disease 2019, G6PD: glucose-6-phosphatase deficiency, DM1: diabetes mellitus type 1

Clinical features	Patient 1	Patient 2	Patient 3	Patient 4	Patient 5	Patient 6	Patient 7	Patient 8	Patient 9	Patient 10	
Fever	+	+	+	+	+	+	+	+	+	+	
Diarrhea	+	-	+	-	+	+	+	+	-	+	
Abdominal pain and emesis	+	+	+	+		+	+	+	+	+	
Conjunctivitis	+	+	-	+	+	+	+	+	+	+	
Rash	+	+	+	+	+	+	+	+	+	+	
Lymphadenopathy	+	+	-	+		+	-	-	+	-	
Cracked lips	+	+	-	+		-	-	+	+	+	
Extremity edema	+	+	-	-		+	-	+	+	-	
Irritability	+	+	+	+	+	+	-	+	-	-	
Shock	+	+	+	+	+	+	+	+	+	+	
COVID-19 exposure	+	+	+	+	+	+	+	+	+	+	
Special	G6PD deficient	-	G6PD deficient	DM1	-	-	Previous Kawasaki	Entamoeba in stool	Influenza b	G6PD deficient	

**Table 2:** Laboratory Data LDH: lactate dehydrogenase, AST: aspartate aminotransferase, ALT: alanine aminotransferase, PT: prothrombin time, PTT: partial thromboplastin time, INR: international normalized ratio, ESR: erythrocyte sedimentation rate, C-RP: C-reactive protein, COVID-19: coronavirus disease 2019, IgG: immunoglobulin G, PCR: polymerase chain reaction

Laboratory Feature	Patient 1	Patient 2	Patient 3	Patient 4	Patient 5	Patient 6	Patient 7	Patient 8	Patient 9	Patient 10
Complete blood count										
Total White blood cell count										
Initial	15.2	3	36.52	10.6	5.56	7.24	4.8	8.66	5.9	3.5
Final	28.2	7.59	19.45	18.49	5.94	21.07	23	5.99	8.1	11.1
Ref : 4.5-13.5 ×103/μL										
Hemoglobin (g/dL)										
Initial	10.2	7.1	7.1	9.2	9.7	9.3	10.9	9.3	9.7	8.4
Final	10.4	10.6	8.5	7.9	8.6	8.2	9.8	9.9	11.7	9.6
Ref :12.5-13.7 g/dL										
Platelets (×103 /μL)										
Initial	170	70	473	182	152	107	151	42	230	96
Final	205	106	587	313	131	284	182	256	1002	561
Ref: 150-350 ×103/μL										
Biochemistry										
calcium										
Initial	1.71	1.82	2.08	1.99	2.08	2.26	1.83	1.65	1.85	1.99
Final	2.1	1.89	2	2.07	2	2.1	1.89	1.99	2.03	2.24
Ref : 2.1-2.5 mmol/L										
Sodium										
Initial	125	132	135	129	127	132	133	126	128	132
Final	137	133	135.4	133.6	133	134	136	135	138	138
Ref: 135-145 mmol/L										
Potassium										
Initial	3	3.56	4.34	3.19	3.2	3.86	3.7	2.46	3.29	3.9
Final	4.1	4.26	3.68	3.435	3.51	3.93	4.2	3.2	3.77	4.7
Ref: 3.5-5 mmol/L										
Magnesium										
Initial	0.7	0.84	0.96	0.73	0.87	0.86	0.77	0.73	0.81	0.72
Final	0.98	1.05	1	0.733	0.61	0.75	0.9	0.67	0.77	0.98
Ref: 0.63-1.05 mmol/L										
Phosphorus										
Initial	0.63	1.4	1.14	1	0.9	1.51	0.73	0.64	0.75	0.84
Final	2.26	1.39	1.29	0.728	0.54	0.95	0.92	0.89	0.64	1.28
Ref: 0.78-1.42 mmol/L										
LDH										
Initial	258	508	508	62	307	379	230	201	197	202
Final	5528	523	296	196	299	Not taken	209	207	228	181
Ref : 100-190 units/L										
Albumin										
Initial	28.7	28.1	25.2	29	28.9	38	34	17	24	27
Final	28	23.5	24	21.7	25.5	28.2	28	29	32	34
Ref : 34.0-50.0 g/L										
AST										
Initial	44	92.2	20.4	3.5	41	40	34	43	31	ND
Final	168	150	12	21.542	13.9	ND	11	23	ND	ND
Ref : 13-35 unit/L										
ALT										
Initial	44	60	4.6	3.4	51	61.3	39	41	12	41
Final	1490	82	4.1	18.27	24.5	Not taken	15	31	ND	ND
Ref : 10-30 unit/L										
Blood Gases:										
pH										
Initial	7.38	7.108	7.43	7.318	7.35	7.36	7.49	7.17	7.53	7.5
Final	7.06	7.045	3.42	7.452	Not taken	Not taken	7.49	7.3	Not taken	7.4
Ref : 7.35-7.45										
Hco3										
Initial	21	12.8	24.8	17.8	19.6	19.3	20	10	23	26
Final	43	14.8	24.5	26.6	Not taken	Not taken	19	27	Not taken	27
Ref : 21-28 mmol/L										
PCo2										
Initial	34	44.1	38.3	34.4	34.9	33.6	27	23	28	34
Final	14	82.5	37.3	37.9	Not taken	Not taken	26	44	Not taken	42
Ref : 35-45 mmHg/L										
Coagulation Profile:										
PT										
highest	24.4	13.9	12.8	14.2	14.3	12.8	19	22	23	16.2
Ref : 11.0-13.5 seconds										
PTT										
highest	78.3	46	41.2	36.3	40.8	42.8	42	55	38	45
Ref : 30-40 seconds										
INR										
highest	2.17	1.22	0.97	1.09	13.3	1.12	1.55	1.66	2	1.28
Ref : 0.8-1.1										
D-dimer	Not available	Not available	7.22	Not available	Not available	Not available	9.9	12.02	3.71	3.84
Ref: <0.50 mg/L										
Inflammatory Markers:										
ESR	101	36	140	73	3	65	40	10	84	18
Ref : 0.0-20 mm/hour										
C-RP	Not available	Not available	Not available	Not available	Not available	Not available	325	202	264	154
Ref: <10 mg/L										
Ferritin										
highest	800	Not taken	1258	Not taken	805	271	430	721	407	1337
Ref : 7-140 ng/mL										
Cardiac enzymes:										
Troponin	0.454	Not taken	Not taken	Not taken	Not taken	Not taken	0.008	0.009	0.037	0.052
Ref : <0.2ng/ml										
COVID-19 tests										
COVID-19 IgG	Positive (70mg/dl)	Not taken	Not taken	Not taken	Not taken	Not taken	Positive	Positive	Positive	Positive
COVID-19 PCR	Positive	Positive	Positive	Negative twice	Positive	Positive	Negative	Positive	Negative	Negative

**Table 3:** Radiological Data CT: computerized tomography, ND: not done

Radiological investigations	Patient 1	Patient 2	Patient 3	Patient 4	Patient 5	Patient 6	Patient 7	Patient 8	Patient 9	Patient 10
X-ray finding	borderline cardiomegaly with clear lung fields	acute respiratory distress picture(bilateral infiltration)	Normal	Normal	Normal	Normal	Normal	right-sided pleural effusion	Normal	Normal
Echocardiography finding	depressed left ventricular function, mitral valve regurgitation, and mild pericardial effusion	pericardial effusion	Normal	Normal	Normal	Normal	Normal	Normal	Left ventricular dysfunction	Normal
Abdominal ultrasound	mesenteric lymphadenitis	hydrops of the gallbladder	Normal	Normal	Normal	Normal	Consistent with acute appendicitis, CT abdomen showed mesenteric adenitis	Mild ascites	Moderate ascites	ND

**Table 4:** Management and Outcome Epi: epinephrine, NorEpi: norepinephrine, Mil: milrinone, Dop: dopamine, IVIG: intravenous immunoglobulin

Management and outcome	Patient 1	Patient 2	Patient 3	Patient 4	Patient 5	Patient 6	Patient 7	Patient 8	Patient 9	Patient 10
Ventilatory support	+	+	-	-		-	-	-	-	-
Vasoactive support	Epi, NorEpi, mil	Epi, NorEpi	-	-	NorEpi	-	-	Epi, Dop	Epi	-
IVIG	+	+	+	+	+	+	+	+	+	+
Antibiotic	+	+	+	+	+	+	+	+	+	+
Steroids	+	+	+	+	+	+	+	+	+	+
Antiviral (Favipiravir)	+	+	+	+	+	+	-	+	-	-
Tocilizumab	+	-	-	-	-	-	-	+	-	-
Heparin	+	+	+	+	+	+	+	+	+	+
Aspirin	-	-	-	-	-		+	+	+	+
Outcome	Death	Death	Recovery	Recovery	Recovery	Recovery	Recovery	Recovery	Recovery	Recovery

## Discussion

We have described 10 patients younger than 14 years of age who met the criteria for MIS-C associated with COVID-19 infection from Al-Ahsa, Saudi Arabia.

MIS-C is an inflammatory syndrome with dermatologic, mucocutaneous, and gastrointestinal manifestations complicated with cardiac dysfunction. This collection of symptoms suggests an inflammatory vasculitis, which shares a lot of similarities between Kawasaki disease and toxic shock syndrome [5]. In our case series, all patients presented with Kawasaki-like disease and some sort of shock-like picture.

The case definition of MIS-C that we used in this case series was based on the following criteria: severe disease leading to hospitalization, age less than 21 years, fever for more than 24 hours, laboratory evidence of inflammation, multisystem organ involvement, and evidence of infection with COVID-19 based on RT-PCR, antibody testing, or exposure to persons with COVID-19 in the past month [6].

We observed more cases of MIS-C in our sites in June and July; this is probably because the peak of cases in Al-Ahsa was recorded in May and June and MIS-C is usually observed as a late reflection of the acute illness of COVID-19.

The vast majority of patients developed significant electrolytes imbalance specifically hyponatremia, and hypokalemia most likely attributed to gastrointestinal loss. Hypocalcemia can be explained by severe inflammation which has unknown mechanism. Hypoalbuminemia was universal in all patients and this probably because of significant systemic inflammation which will lead to leakage of albumin from blood vessels to tissues.

One of the interesting observations in our findings that three patients (30%) had underlying G6PD deficiency, which is very prevalent in Al-Ahsa, Saudi Arabia (23% prevalence rate) [7]. Wu et al. wrote that G6PD-deficient cells are more vulnerable to human coronavirus infection than G6PD-normal cells [8] but we do not know if G6PD deficiency is a risk factor for severe COVID-19 disease or severe MIS-C.

Another interesting finding is that one of the patients had recurrent Kawasaki disease, first at 4 months with an unknown triggering factor, and this time at the age of 12 years triggered by COVID-19 infection.

As more is learned about MIS-C and COVID-19, it is becoming apparent that there is a wide spectrum of disease severity [9].

With approximately more than 2000 cases of MIS-C reported worldwide, clinicians are still facing difficulties in diagnosing and treating MIS-C patients [9]. There are many unanswered questions about MIS-C: what is the incidence and prevalence of this disease? What is the pathogenesis? Whom are at risk of developing the disease and why? Any risk factor of developing severe disease? What treatments may prevent progression to shock, or why they progress to shock? Will treatment prevent coronary-artery aneurysms? To answer these questions, we need further investigation by large-scale in-depth studies [4].

## Conclusions

There are a lot of unknowns about COVID-19 and MIS-C. Meanwhile, clinicians should be very alert and they should raise the index of suspicion about MIS-C in patients recovered from COVID-19 infection. Because MIS-C patients can deteriorate quickly, we recommend starting management as early as possible.
